# The efficacy assessment of convalescent plasma therapy for COVID-19 patients: a multi-center case series

**DOI:** 10.1038/s41392-020-00329-x

**Published:** 2020-10-06

**Authors:** Hao Zeng, Dongfang Wang, Jingmin Nie, Haoyu Liang, Jiang Gu, Anne Zhao, Lixin Xu, Chunhui Lang, Xiaoping Cui, Xiaolan Guo, Changlong Zhou, Haibo Li, Bin Guo, Jinyong Zhang, Qiang Wang, Li Fang, Wen Liu, Yishan Huang, Wei Mao, Yaokai Chen, Quanming Zou

**Affiliations:** 1 National Engineering Research Center of Immunological, Department of Microbiology and Biochemical Pharmacy, College of Pharmacy and Laboratory Medicine, Third Military Medical University, Chongqing, P.R. China; 2 State Key Laboratory of Trauma, Burn and Combined Injury, Third Military Medical University, Chongqing, P.R. China; 3 Institute of Blood Transfusion, Chongqing Blood Center, Chongqing, P.R. China; 4 Chongqing Public Health Medical Central, Chongqing, P.R. China; 5 Division of HIV/AIDS and Sex-transmitted Virus Vaccines, Institute for Biological Product Control, National Institutes for Food and Drug Control, Beijing, P.R. China; 6 Chongqing University Three Gorges Hospital & Chongqing Three Gorges Central Hospital, Chongqing, P.R. China; 7 Affiliated Hospital of North Sichuan Medical College, Nanchong, P.R. China; 8 Yongchuan Hospital of Chongqing Medical University, Chongqing, P.R. China

**Keywords:** Infectious diseases, Immunotherapy, Immunology

## Abstract

Convalescent plasma (CP) transfusion has been indicated as a promising therapy in the treatment for other emerging viral infections. However, the quality control of CP and individual variation in patients in different studies make it rather difficult to evaluate the efficacy and risk of CP therapy for coronavirus disease 2019 (COVID-19). We aimed to explore the potential efficacy of CP therapy, and to assess the possible factors associated with its efficacy. We enrolled eight critical or severe COVID-19 patients from four centers. Each patient was transfused with 200–400 mL of CP from seven recovered donors. The primary indicators for clinical efficacy assessment were the changes of clinical symptoms, laboratory parameters, and radiological image after CP transfusion. CP donors had a wide range of antibody levels measured by serology tests which were to some degree correlated with the neutralizing antibody (NAb) level. No adverse events were observed during and after CP transfusion. Following CP transfusion, six out of eight patients showed improved oxygen support status; chest CT indicated varying degrees of absorption of pulmonary lesions in six patients within 8 days; the viral load was decreased to a negative level in five patients who had the previous viremia; other laboratory parameters also tended to improve, including increased lymphocyte counts, decreased C-reactive protein, procalcitonin, and indicators for liver function. The clinical efficacy might be associated with CP transfusion time, transfused dose, and the NAb levels of CP. This study indicated that CP might be a potential therapy for severe patients with COVID-19.

## Introduction

In December 2019, the outbreak of coronavirus disease 2019 (COVID-19) caused by severe acute respiratory syndrome coronavirus 2 (SARS-CoV-2) emerged in Wuhan, China, and has rapidly spread around the world.^1^ COVID-19 can manifest on a spectrum of illness from mild disease to severe respiratory failure requiring intensive care unit admission. The World Health Organization (WHO) has declared COVID-19 a pandemic on March 11, 2020. As of May 17, 2020, it had caused a total of 4,525,497 cases of infection and resulted in 307,395 deaths globally.^2^

Epidemiology and etiology for COVID-19 are rapidly evolving, giving us a greater understanding of those at risk and elucidating more potential therapy targets.^3^ In addition to supportive care, such as oxygen support and extracorporeal membrane oxygenation, several drugs for this disease are still being researched, such as remdesivir, lopinavir/ritonavir, arbidol, and darunavir.^4,5^ However, up to now, no approved vaccine or specific antiviral agents has been proved to be effective to prevent or treat SARS-CoV-2 infection due to the absence of evidence.

Passive immunity delivered as neutralizing antibodies (NAbs) from convalescent plasma (CP) may offer an alternative therapeutic approach for COVID-19.^6^ CP therapy has been empirically used in other epidemics, including SARS, Middle East respiratory syndrome (MERS), and 2009 influenza A (H1N1).^7–10^ A meta-analysis of 32 reports on SARS coronavirus infection and severe influenza revealed a statistically significant reduction of mortality after administration of CP, especially when CP was given early after symptom onset.^8^ However, in a case series on influenza A (H5N1) virus infection, nonsignificant benefits following the intervention of CP were reported,^11^ and no association of CP therapy with an increased survival was observed in 84 patients with Ebola virus disease.^12^ It is possibly due to the unknown levels of NAbs in the infused plasma, which may obscure the effects of CP.^10^ In this current pandemic, preliminary studies suggested the effectiveness of CP with no severe adverse events to treat patients infected with SARS-CoV-2.^13–17^ The results from a pilot study applying CP transfusion for 10 severe patients showed that administration of CP with NAb titers above 1:640 led to improvement in clinical symptoms and pulmonary lesions.^14^ These findings indicate that CP transfusion may be a promising therapy in the treatment for COVID-19.

Nonetheless, due to the limitations of the study design and small sample size, current evidence on the efficacy and safety of CP therapy for COVID-19 is still limited. Moreover, the quality control of CP and individual variation in patients in different studies make it rather difficult to evaluate the efficacy and risk of CP therapy. Thus, more supporting evidence (such as multi-level assessment of specific antibodies in CP, indications for CP treatment, and selection of transfusion timing) is called for with wider adoption of CP for COVID-19 in multi-centers and regions. Herein, we performed a retrospective observational study involving eight critical or severe patients with COVID-19 from four designated hospitals in the southwest region of China, aiming to explore the potential efficacy and safety of CP therapy, and to provide more evidence for the quality control of donated plasma and reasonable clinical application of CP transfusion.

## Results

### Clinical characteristics of the patients

A total of 8 patients (4 males and 4 females) with critical or severe COVID-19 were enrolled. The median age was 65.0 years (IQR, 63.0–67.0 years). The median time from symptom onset to hospital admission was 4.0 days (interquartile interval (IQR), 3.0–8.5 days). The most common symptoms during hospitalization were cough (7/8), shortness of breath (5/8), and fever (4/8), while patients had fewer manifestations of dyspnoea (two cases), diarrhea (two cases), headache (one case), and fatigue (one case). Five patients had coexisting chronic diseases at admission, including type II diabetes, hypertension, chronic obstructive pulmonary disease (COPD), and coronary heart disease (CHD).

Table 1 listed the drug treatments prior to and after CP transfusion. All 8 patients received combination therapy of various antiviral treatment and other supportive care. The most commonly used antivirals were interferon alfa-1b (8/8), lopinavir/ritonavir (7/8), and arbidol (7/8). Darunavir and hydroxychloroquine sulfate were also administered for three and two patients, respectively. Antibiotic or antifungal agents were used when patients had co-infection. Five patients were given corticosteroids at the appropriate situation. Chest computed tomography (CT) scans demonstrated that all patients presented bilateral multiple ground-glass opacity or partial consolidation at the time of admission, with primary involvement of subpleural lesions.

**Table 1:** Basic clinical characteristics of patients with COVID-19

	Patients							
	1	2	3	4	5	6	7	8
Centers	1	1	1	2	3	4	4	4
Age	70	64	46	60	84	66	65	65
Sex	Female	Female	Female	Male	Male	Female	Male	Male
Smoking history	None	None	None	None	None	None	None	Current smoking
Estimated incubation period, days	4	None	None	10	10	6	-	2
Days from symptom onset to admission	7	3	4	10	3	2	-	13
Comorbidities	Type II diabetes	None	None	Hypertension	COPD	None	PD	Type II diabetes, CHD, COPD
Principal symptoms during hospitalization	Cough, sputum production, shortness of breath, diarrhea	Cough, shortness of breath, diarrhea	Cough, dyspnoea, shortness of breath	Fever, cough, dyspnoea	Fever, cough, shortness of breath	Fever, headache, shortness of breath	Confusion, static tremor, cough, sputum production	Fever, cough, sputum production, fatigue
Treatments								
Antivirals	Lopinavir/ritonavir, arbidol^a^, darunavir^a^, interferon alfa-1b^a^	Lopinavir/ritonavir, arbidol^a^, darunavir^a^, interferon alfa-1b	Lopinavir/ritonavir, interferon alfa-1b, arbidol, darunavir	Arbidol, interferon alfa-1b	Lopinavir/ritonavir, arbidol, interferon alfa-1b	Lopinavir/ritonavir, ribavirin, hydroxychloroquine sulfate^a^, interferon alfa-1b^a^	Lopinavir/ritonavir, arbidol, interferon alfa-1b	Lopinavir/ritonavir, arbidol^a^, interferon alfa-1b, hydroxychloroquine sulfate, lamivudine
Antibiotics or antifungal agents	Moxifloxacin	None	None	Levofloxacin, potassium amoxicillin clavulanate, linezolid, voriconazole^a^, tigecycline^a^, Piperacillin-tazobactam^a^, aztreonam^a^	Piperacillin-tazobactam, caspofungin	Moxifloxacin^a^, piperacillin-tazobactam	Moxifloxacin, cefoperazone sodium and sulbactam sodium^a^, metronidazole^a^	Voriconazole, cefoperazone sodium and sulbactam sodium, moxifloxacin, meropenem, piperacillin-tazobactam, imipenem and cilastatin sodium
Corticosteroids	None	None	Methylprednisolone	Methylprednisolone	Methylprednisolone^a^	Methylprednisolone^a^	None	Methylprednisolone^a^
Others	Thymosin^a^	Thymosin^a^	Thymosin	Immunoglobulin, ambroxol	Thymosin^a^, gamma globulin^a^	Thymosin	Thymosin, xuebijing injection	Thymosin^a^

*COPD* chronic obstructive pulmonary disease, *PD* parkinson’s disease, *CHD* coronary heart disease

^a^Regarding the drugs administered after the CP transfusion within 5 days. Center 1–4 were Chongqing Public Health Medical Center, Affiliated Hospital of North Sichuan Medical College, Yongchuan hospital of Chongqing Medical University, and Chongqing Three Gorges Central Hospital, respectively

### Characteristics of convalescent plasma donors

In total, seven donors (5 males and 2 females) from the participating hospitals who had recovered from SARS-CoV-2 infection donated 300–400 mL of CP (Table 2). The median age was 37.0 years (IQR, 34.0–42.5 years). These donors donated the CP at the median day of 11.0 (IQR, 9.5–17.5 days) from discharge. All of 7 donors were mild or moderate patients during a hospital stay with no comorbidities.

**Table 2:** Characteristics of convalescent plasma donors

	Donors						
	1	2	3	4	5	6	7
Blood products coding	399	400	397	933	395	703	701
Centers	1	1	1	2	1	3	3
Sex	Male	Female	Male	Male	Female	Male	Male
Age	26	49	39	41	47	34	43
Donated plasma volume, mL	400	400	400	300	400	400	400
Blood type	A	A	A	A	B	A	B
Clinical classification	Mild	Moderate	Mild	Mild	Moderate	Moderate	Moderate
Comorbidities	None	None	None	None	None	None	None
Estimated incubation period, d	–	20	3	–	3	9	6
Days of plasma donation from symptom onset	16	19	30	30	33	19	22
Anti-SARS-CoV-2 specific IgM MCLIA titer	<1:50	1:50	<1:50	1:320	<1:50	1:50	1:50
Anti-SARS-CoV-2 specific IgG MCLIA titer	1:160	1:160	1:320	1:1280	1:1280	1:160	1:1280
Anti-S-RBD specific IgG ELISA titer	1:640	1:640	1:1280	1:2560	1:2560	1:1280	1:2560
Anti-NP specific IgG ELISA titer	1:640	1:320	1:1280	1:2560	1:2560	1:2560	1:5120
IT50	1:6	1:3	1:74	1:33	1:12	1:17	1:20
NAT50	1:320	1:255	1:312	1:1529	1:852	1:460	1:1576

*MCLIA* magnetic chemiluminescence enzyme immunoassay, *ELISA* enzyme-linked immunosorbent assay, *S-RBD* receptor binding domains of spike protein, *NP* nucleoprotein, *IT50* inhibitory titer which was calculated with the dilution of plasma that inhibits 50% RBD-Fc binding to receptor ACE2, *NAT50* neutralizing antibody titer which was calculated with the highest dilution of plasma that resulted in a 50% reduction of virus infection

Center 1–3 were Chongqing Public Health Medical Center, Affiliated Hospital of North Sichuan Medical College, and Chongqing Three Gorges Central Hospital, respectively

We measured SARS-CoV-2 specific antibodies using four platforms of immunological tests. The SARS-CoV-2 specific antibody titers were detected by magnetic chemiluminescence enzyme immunoassays (MCLIA) which targeted at the combination of nucleoprotein (NP) and receptor binding domains of spike protein (S-RBD) specific antigens, as well as by enzyme-linked immunosorbent assays (ELISA) which determined anti-NP and anti-S-RBD specific IgG antibodies separately. The IgG titers detected by MCLIA ranged from 1:160 to 1:1280, and the IgM MCLIA titers were less than or equal to 1:50 in six donors, except donor 4 (1:320). The ELISA results showed that the anti-S-RBD and anti-NP specific IgG titers were in a range of 1:640–1:2560 and 1:320–1:5120, respectively. We measured the inhibitory activity of receptor binding (RBIA) of the CP samples by a receptor-binding assay, finding the 50% inhibitory titer (IT50) values ranging from 1:3 to 1:74. Importantly, the neutralizing activity of these plasma samples, which offer the most informative assessment of antiviral activity of patient sera against viral infection, was measured by a pseudovirus based neutralization assay. The NAbs of the donated plasma also showed variable levels (NAb titer (NAT50) range, 1:255–1:1576), and only three CP donors (donor 4, 5, and 7) had NAT50 values greater than 1:640.

**Fig. 1:**
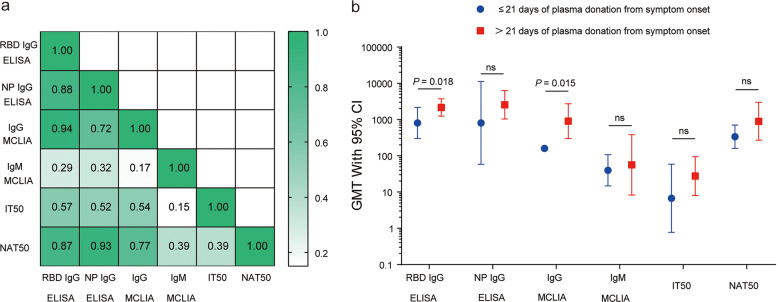
SARS-CoV-2 specific antibody levels of CP samples measured by serology tests, receptor-binding assay, and pseudovirus based neutralization assay. **a** The correlations among anti-SARS-CoV-2 specific IgG and IgM titers detected by commercial MCLIA kits, anti-S-RBD and anti-NP specific IgG titers determined by in-house ELISA assays, inhibition activity measured by a receptor-binding assay, and neutralizing antibody titer measured by a pseudovirus based neutralization assay. **b** Comparisons of antibody levels between CP samples collected before and after 21 days from symptom onset. MCLIA magnetic chemiluminescence enzyme immunoassay, ELISA enzyme-linked immunosorbent assay, RBD receptor binding domains, NP nucleoprotein, IT50 inhibitory titer which was calculated with the dilution of plasma that inhibits 50% RBD-Fc binding to receptor ACE2, NAT50 neutralizing antibody titer which was calculated with the highest dilution of plasma that resulted in a 50% reduction of virus infection, GMT geometric mean titer, CI confidence interval

The results of correlation analyses as shown in Fig. 1a indicated that there was positive correlation between IgG MCLIA titer and S-RBD specific IgG ELISA titer (*r* = 0.94, *P* = 0.029). NAT50 was positively correlated with S-RBD and NP specific IgG ELISA titers, respectively (*r* = 0.87, *P* = 0.019; *r* = 0.93, *P* = 0.007, respectively). However, the positive association between IgG MCLIA titer and NAT50 did not show statistical significance (*r* = 0.77, *P* = 0.071). Notably, IT50 was neither related to NAT50, nor correlated to IgG titers. Comparing the antibody levels of CP collected at different time, we found that the CP donated greater that 21 days had higher levels of S-RBD IgG ELISA titer and IgG MCLIA titer than CP which collected less than or equal to 21 days (Fig. 1b).

### CP treatment

The detailed information about CP treatment for the 8 patients were shown in Table 3. These patients were administered one or two transfusions of CP. Two transfusions were administered with an interval less than 24 h. ABO-compatible and cross-matched CP were administered at the discretion of the attending clinicians and according to plasma availability. Patients received CP transfusion between 9 and 34 days following the onset of symptoms, with three of them given within 21 days from symptom onset. Five of eight patients received two doses of 100–200 mL of CP within 24 h (totally 300 or 400 mL), while the other three cases only received one dose of 200 mL.

**Table 3:** Detailed information about patients receiving convalescent plasma treatment

	Patients							
	1	2	3	4	5	6	7	8
Complications prior to CP treatment	Bacterial pneumonia	Hyperlipidaemia	None	Bacterial pneumonia, ARDS, MODS, LDVT	Fungal pneumonia, cardiac failure, anemia	None	Bacterial pneumonia	Fungal pneumonia, LDVT
Clinical classification prior to CP treatment	Severe	Critical	Severe	Critical	Severe	Severe	Severe	Severe
Days of CP transfusion from symptom onset	30	12	17	23	34	26	>9	28
Total transfusion volume, mL	400	400	400	300	400	200	200	200
1st transfusion								
CP donor	1	2	3	4	5	6	7	6
Time	Feb 18, 00:10 a.m.	Feb 18, 00:15 a.m.	Feb 29, 10:00 a.m.	Feb 20, 14:05 p.m.	Mar 5, 20:10 p.m.	Feb 23, 13:20 p.m.	Feb 23, 13:10 p.m.	Feb 23, 13:10 p.m.
Dose, mL	200	200	200	200	200	200	200	200
2nd transfusion								
CP donor	2	1	3	4	5	–	–	–
Time	Feb 18, 12:50 p.m.	Feb 18, 12:50 p.m.	Mar 1, 10:05 a.m.	Feb 21, 15:05 p.m.	Mar 6, 8:45 a.m.	–	–	–
Dose, mL	200	200	200	100	200	–	–	–
Clinical outcome	Discharge	Discharge	Discharge	Remain hospitalized	Discharge	Discharge	Discharge	Discharge
Length of hospital stay, days	36	20	18	-	35	28	28	30

*CP* convalescent plasma, *ARDS* acute respiratory distress syndrome, *MODS* multiple organ dysfunction syndrome, *LDVT* deep vein thrombosis in lower limb

### Clinical response of CP transfusion

#### Adverse Effects of CP Transfusions

No adverse events were observed in the eight patients after CP transfusion.

#### Clinical characteristics

**Fig. 2:**
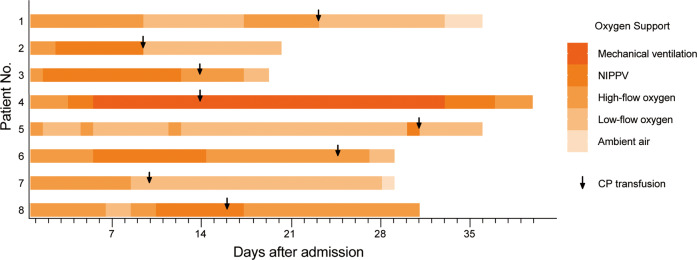
Changes in oxygen-support status from admission in individual patients. For each patient, the colors in the line represent the oxygen-support status of the patient over time. Invasive ventilation includes invasive mechanical ventilation. Noninvasive ventilation includes noninvasive positive pressure ventilation (NIPPV), high-flow oxygen therapy with nasal or face mask, and low-flow oxygen therapy with nasal. The vertical black arrows show the day of CP transfusion. The presentation of oxygen support status referred to a recent report^5^

As the patients have been treated by antiviral drugs and oxygen support before CP therapy, the body temperature, heart rate, and systolic pressure were normal even prior to CP transfusion, and kept unchanged within 5 days after CP transfusion as indicated in Table 4. Individual patient’s change in the category of oxygen support during hospitalization are shown in Fig. 2. Six of eight patients showed an improvement in the category of oxygen support within 5 days from CP treatment. Obvious improvement was observed in patients who were receiving high-flow nasal cannula oxygenation (*n* = 3), or noninvasive positive pressure ventilation (NIPPV, *n* = 3) prior to CP treatment. It is notable that patient 1, 2, and 5 rapidly shifted high-flow supplemental oxygen or NIPPV to low-flow supplemental oxygen within 24 h after CP transfusion.

**Table 4:** Clinical features and laboratory results before and after convalescent plasma transfusion

	After CP transfusion					
	Before CP transfusion	Day 1	Day 2	Day 3	Day 4	Day 5
*Clinical characteristics*						
Body temperature, °C	36.5 (36.3–36.6)	36.6 (36.2–36.9)	36.6 (36.3–36.9)	36.6 (36.3–36.8)	36.5 (36.3–36.8)	36.4 (36.3–37.1)
Respiratory rate, per min	19.5 (19.0–25.5)	21.0 (20.0–23.5)	20.5 (20.0–21.0)	21.5 (19.0–22.0)	20.0 (19.3–21.8)	20.0 (19.0–25.0)
Heart rate	77.5 (64.0–92.5)	81.0 (72.0–87.8)	78.0 (62.5–88.0)	84.5 (73.3–91.5)	87.0 (79.8–89.8)	100.0 (81.5–121.5)
Systolic pressure	118.0 (105.0–129.0)	112.5 (106.8–119.5)	116.0 (114.5–128.0)	116.5 (109.0–123.3)	108.0 (106.0–116.0)	118.0 (109.5–125.8)
PaO_2_/FiO_2_ (normal range, 400–500 mmHg)	259.0 (163.0–283.0)	312.0 (246.0–409.0)	318.0 (279.5–416.0)	225.0 (194.5–444.5)	290.0 (196.0–493.5)	326.0 (215.3–556.0)
*Laboratory results*						
WBC count × 10^9^/L (normal range, 3.5–9.5)	7.5 (5.5–8.4)	8.3 (7.0–9.2)	7.4 (5.5–7.9)	6.6 (5.5–9.9)	6.7 (5.8–10.9)	7.9 (5.6–13.2)
NE count × 10^9^/L (normal range, 1.8–6.3)	6.1 (4.4–7.3)	6.9 (4.5–8.4)	5.7 (4.7–6.9)	5.0 (4.2–8.4)	4.5 (3.5–9.0)	6.0 (3.5–11.8)
LY count × 10^9^/L (normal range, 1.1–3.2)	0.4 (0.2–1.5)	0.7 (0.3–1.2)	0.8 (0.2–1.2)	0.8 (0.4–1.3)	1.1 (0.5–1.5)	1.1 (0.7–1.4)
CRP, mg/L (normal range, <8)	10.9 (7.7–94.0)	11.7 (8.1–51.4)	41.7 (9.5–66.8)	24.9 (6.3–107.4)	24.1 (6.1–95.2)	41.9 (5.0–58.1)
PCT, ng/mL (normal range, <0.1)	0.15 (0.06–0.38)	0.09 (0.05–0.18)	0.05 (0.05–0.13)	0.04 (0.03–0.12)	0.04 (0.01–0.09)	0.01 (0.01–0.11)
IL-2, pg/mL (normal range, 0–5.71)	1.1 (0.6–1.6)	0.9 (0.6–0.9)	0.8 (0.6–3.6)	1.3 (1.0–2.1)	0.5 (0.4–1.3)	1.0 (0.5–2.7)
IL-4, pg/mL (normal range, 0–2.80)	1.6 (0.7–2.1)	0.5 (0.3–2.4)	1.4 (0.7–3.3)	1.5 (0.4–2.2)	0.4 (0.2–1.1)	0.7 (0.6–5.1)
IL-6, pg/mL (normal range, 0–5.30)	7.8 (1.8–26.8)	10.5 (1.1–12.8)	12.7 (5.1–32.8)	11.6 (4.3–68.0)	23.6 (4.3–58.3)	6.6 (5.5–35.6)
IL-10, pg/mL (normal range, 0–4.91)	3.1 (2.6–3.7)	3.3 (2.3–10.9)	3.6 (2.5–6.9)	3.6 (3.5–3.7)	3.5 (2.7–3.9)	3.8 (3.3–4.1)
IL-17A, pg/mL (normal range, 0–20.60)	8.2 (0.7–17.0)	1.5 (0.7–8.3)	1.5 (0.0–24.5)	14.0 (3.4–26.7)	8.2 (0.0–12.7)	12.3 (1.5–52.0)
TNF-α, pg/mL (normal range, 0–2.31)	3.3 (0.6–3.7)	1.5 (1.3–5.5)	3.2 (1.2–5.8)	2.4 (1.2–4.6)	1.2 (0.9–2.1)	2.3 (1.4–5.8)
IFN-γ, pg/mL (normal range, 0–7.42)	1.4 (1.3–2.8)	2.7 (1.0–4.7)	1.5 (1.1–3.7)	2.3 (1.3–3.6)	1.3 (1.0–2.4)	2.2 (0.6–4.4)
IL-6/IL-10	1.4 (0.6–4.0)	0.7 (0.3–1.4)	4.2 (2.0–13.3)	2.3 (1.2–10.1)	NA	1.7 (1.5–4.2)
IL-6/IL-4	4.5 (2.0–4.7)	12.4 (5.6–19.7)	9.6 (3.7–46.9)	19.3 (4.3–50.3)	NA	9.5 (6.9–20.9)
ALT, μ/L (normol range, 5–40)	21.1 (14.3–47.0)	22.0 (17.0–49.5)	22.0 (14.5–49.1)	23.0 (18.0–51.7)	20.4 (15.8–40.2)	27.8 (14.0–65.0)
AST, μ/L (normol range, 13–35)	20.2 (16.3–30.8)	19.0 (16.4–25.0)	18.0 (15.5–30.3)	18.0 (16.0–32.3)	17.0 (15.3–24.0)	18.4 (14.0–33.6)
TBIL, μmol/L (normal range, 0–26)	17.5 (7.1–24.7)	10.4 (6.6–15.4)	10.4 (5.8–15.8)	12.0 (9.2–14.2)	10.4 (6.3–16.6)	10.8 (9.6–24.6)
PT, second (normal range, 11–14)	11.3 (10.6–12.4)	11.3 (10.1–13.3)	10.6 (9.5–12.9)	10.4 (9.6–13.9)	10.5 (9.6–13.4)	10.9 (10.1–12.9)
D-dimer, mg/mL (normal range, 0–0.05)	1.2 (0.5–4.0)	1.0 (0.4–5.7)	0.9 (0.6–5.3)	0.8 (0.6–5.4)	1.1 (0.6–4.2)	1.2 (0.8–3.3)

Data are indicated as median (IQR)

*CP* convalescent plasma, *WBC* white blood cell, *NE* neutrophil, *LY* lymphocyte, *CRP* C-reactive protein, *PCT* Procalcitonin, *IL* interleukin, *TNF* tumor necrosis factor, *IFN* interferon, *ALT* alanine aminotransferase, *AST* aspartate aminotransferase, *PT* prothrombin time, *NA* not applicable

#### Pulmonary lesions on chest CT examinations

**Fig. 3:**
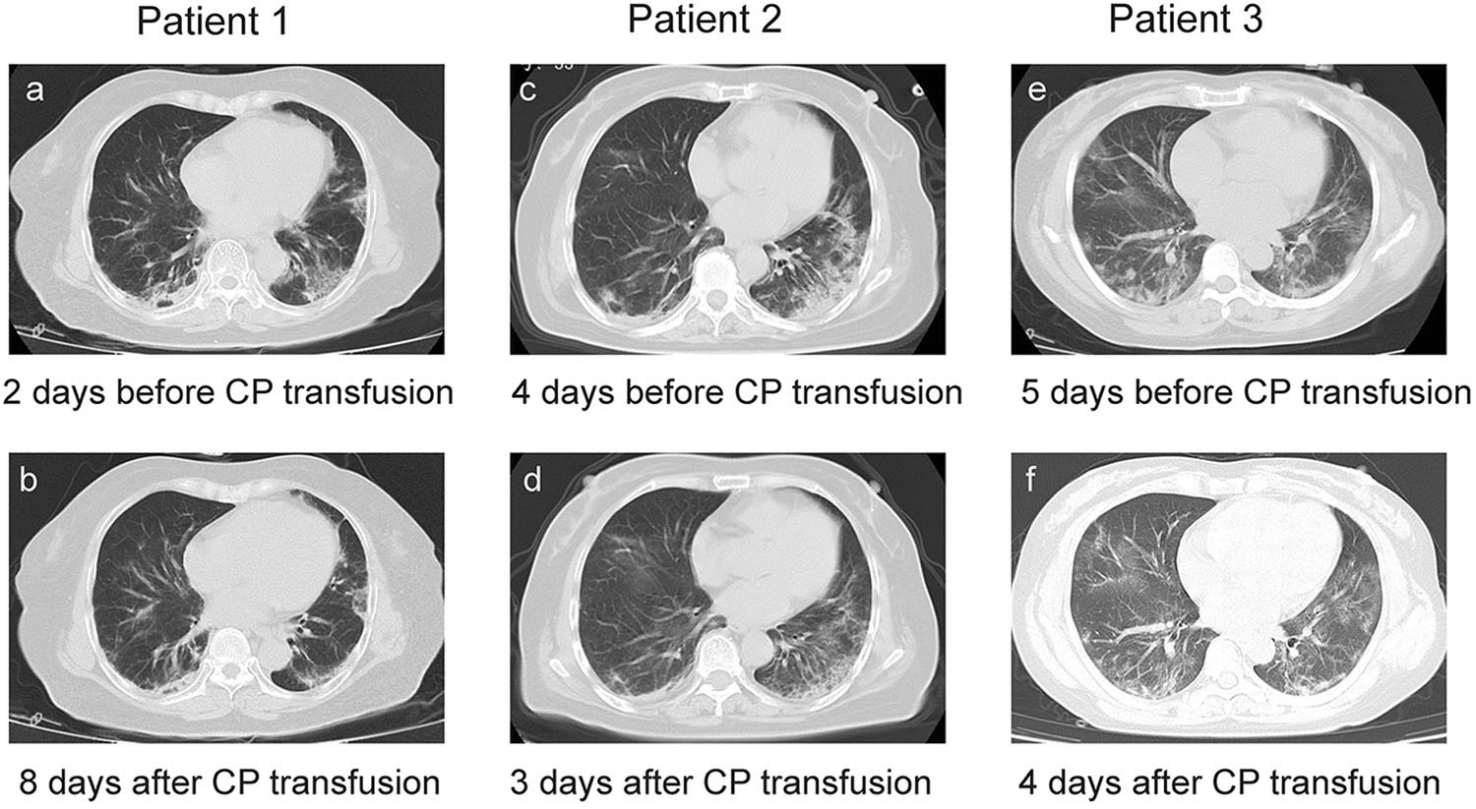
Chest CT scans of three patients. **a** Chest CT of patient 1 obtained on February 16 before CP transfusion (February 18) showed ground-glass opacity with uneven density, close to the pleura. **b** CT Image of patients 1 taken on February 26 showed partial absorption of bilateral ground-glass opacity. **c** Chest CT of patient 2 obtained on February 14 before CP transfusion (February 18) showed diffuse ground-glass opacity in both lungs. **d** CT Image of patients 2 taken on February 21 showed those lesions improved after CP transfusion. **e** CT Image of patients 3 taken on February 24 showed diffusion of bilateral ground-glass opacity before CP transfusion (February 29). **f** CT Image of patients 3 taken on March 4 showed those lesions improved after CP transfusion

Chest CT scans showed that pulmonary lesions improved at varying degrees in six out of eight patients. A partial resolution of pulmonary lesions was observed in patient 2, 3, and 4 on 1st day, in patient 6 and 7 on 3rd day, in patient 4 on 5th day, and in patient 1 on 8th day after plasma transfusion, respectively. Representative chest CT images of patient 1–3 were shown on Fig. 3.

#### Laboratory results

**Fig. 4:**
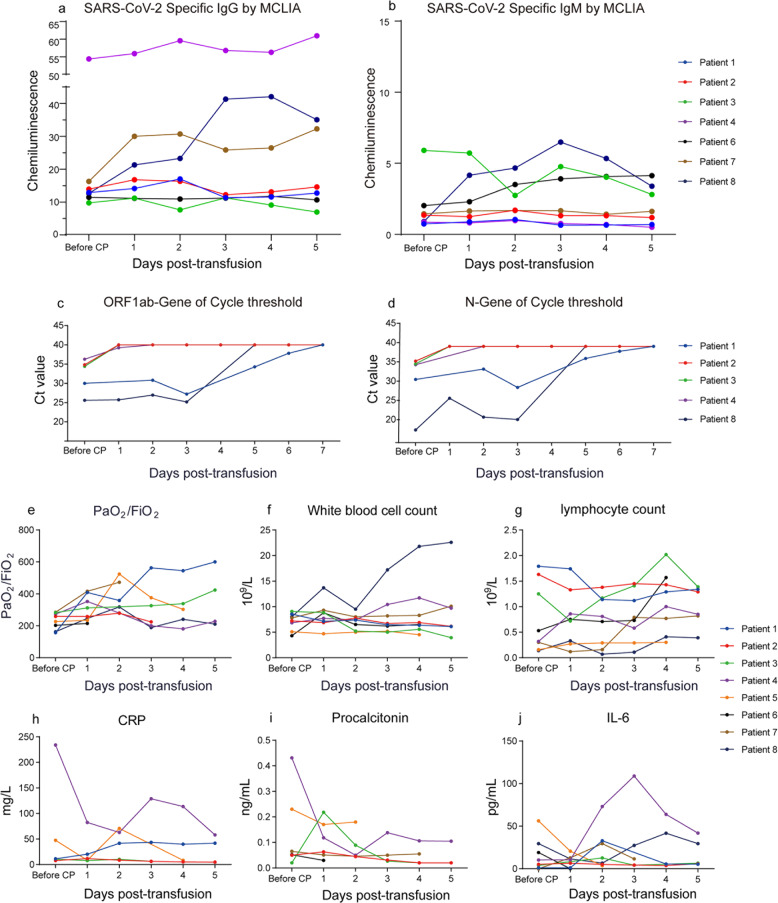
Changes of laboratory results before and at day 1–5 after convalescent plasma transfusion. **a**, **b** SARS-CoV-2 specific IgG and IgM levels, respectively, determined by MCLIA. **c**, **d** Cycle threshold (Ct) values of ORF1ab-gene and N-gene, respectively. A Ct value of 40 was defined as undetectable. **e** PaO_2_/FiO_2_ (normal range: 400–500 mmHg). **f** White blood cell count (normal range: 3.5–9.5). **g** Lymphocyte count (normal range: 1.1–3.2). **h** C-reactive protein (normal range: <8). **i** Procalcitonin (normal range: <0.1). **j** IL-6 (normal range: 0–5.30)

We monitored the development of the virus-specific IgG and IgM antibodies by MCLIA prior to and after CP transfusion in all patients except patient 5. In 5 of 7 patients, the IgG titer increased within 2 days posttransfusion, with patient 4, 7, and 8 presenting the most obvious increment (Fig. 4a). The IgM level was observed lower than IgG for all patients, and waved in a small range after CP transfusion (Fig. 4b).

SARS CoV-2 viral load, estimated by the cycle threshold (Ct) value from reverse transcriptase-polymerase chain reaction (RT-PCR), was positive in five patients before CP transfusion (for other three cases, the data of Ct values was not available). The Ct value was decreased to a negative level in patient 2 and 3 on posttransfusion day 1, patient 4 on day 2, patient 8 on day 5, and patient 1 on day 7 (Fig. 4c, d), which was basically consistent with the improvement of pulmonary lesions indicated by CT scans mentioned above.

The result of arterial blood gas analysis showed that the ratio of the partial pressure of arterial oxygen (PaO_2_) to fraction of inspired oxygen (FiO_2_) (PaO_2_/FiO_2_) (median, 259.0; IQR, 163.0–283.0) prior to transfusion immediately increased one day after transfusion (median, 312.0; IQR, 246.0–409.0), and five patients were indicated a tendency of improvement of PaO_2_/FiO_2_ in the following 5 days after CP therapy (Table 4 and Fig. 4e). Lymphocytopenia, which is a prominent feature of critically ill patients with COVID-19, was also observed in this study, with the median lymphocyte counts of 0.4 (IQR, 0.2–1.5) (Table 4). Within 5 days following plasma transfusion, the lymphocyte counts showed an increase in 6 out of 8 patients (Fig. 4g). The changes of white blood cell count (Fig. 4f) and neutrophil count were similar with an overall downward trend, except that patient 4, 7, and 8, who had the complications of bacterial or fungal pneumonia, presented an increase after CP therapy. As for the inflammatory biomarkers, the increased C-reactive protein (CRP) and procalcitonin before plasma transfusion were observed a declining trend following CP treatment for 4 out of 5 patients, and for 5 of 6 patients, respectively (Fig. 4h, i). Proinflammatory cytokines, including interleukin-6 (IL-6) and tumor necrosis factor-α (TNF-α) demonstrated an increase for 5 of 8 patients (Fig. 4j), and for 5 of 6 patients (Supplementary Fig. S1a), respectively, as compared to the status before CP therapy. Other inflammatory cytokines, such as interferon-γ (IFN-γ), IL-2, IL-10, and IL-17A, showed various alterations in each patient after CP treatment (Supplementary Fig. S1b-e). We also observed tendencies of increment of the ratios of proinflammatory cytokines and anti-inflammatory cytokine (IL-6/IL-10, and IL-6/IL-4) in four patients (Table 4). Concerning the parameters indicative of liver function, the alanine aminotransferase (ALT), aspartate aminotransferase (AST), and total bilirubin (TBIL) tended to decrease after CP therapy, except for an increase of all these indicators in patient 4, and elevated ALT and AST in patient 8. The coagulation profile of patients was also monitored following CP treatment, indicating that 5 out of 6 patients kept the normal level of prothrombin time, while abnormally elevated D-dimer prior to plasma transfusion (median, 1.2; IQR, 0.5–4.0) still increased within 5 days after plasma treatment in 4 of 6 patients (Supplementary Fig S1f).

#### Outcome of patients treated with CP

All patients were discharged from the hospital with a median length of stay of 28.0 days (IQR, 24–32.5 days), except for patient 4, who remained hospitalized for further treatment of underlying diseases as of April 8, 2020.

#### Assessment of possible factors associated with clinical effects

To assess the factors which might affect the clinical effects, we compared the clinical features between patients who received CP transfusion on different time, with different doses, and with different NAb titers (Table 5). Although the results were not statistically analyzed due to limited samples in each group, patients who received CP transfusion before 21 days from symptom onset tended to show a more rapid negative conversion of viral nucleic acid, and shorter hospital stays compared to patients who were transfused after 21 days. Concerning the doses of plasma, we found that the viral nucleic acid in patients transfused with 400 mL of CP had a tendency to turn faster to a negative level than that in patients who received 200 mL of CP. When comparing to patients treated by CP with NAT50 ≤ 1:640, the viral RNA tended to be decreased to an undetectable level in less time, and the increment of IgG MCLIA level indicated by the IgG titer ratio (the titer at day 1 after CP transfusion divided by the value before CP transfusion) tended to be higher in patients receiving CP with NAT50 > 1:640. But the hospitalization was longer for patients receiving high NAT50, mainly because of patient 4 who remained hospitalized for treating for severe complications including acute respiratory distress syndrome (ARDS), multiple organ dysfunction syndrome (MODS), and deep vein thrombosis in lower limb (LDVT).

**Table 5:** Comparison of clinical features between patients who received CP transfusion on different time, with different doses, and with different NAb titers

	Days from symptom onset to CP transfusion (*n* = 8)		Dose of CP transfusion (*n* = 7)		NAT50 of CP (*n* = 8)	
	≤21 day (*n* = 3)	>21 day (*n* = 5)	400 mL (*n* = 4)	200 mL (*n* = 3)	>1:640 (*n* = 3)	≤1:640 (*n* = 5)
Patient No.	Patient 2 Patient 3 Patient 7	Patient 1 Patient 4 Patient 5 Patient 6 Patient 8	Patient 1 Patient 2 Patient 3 Patient 5	Patient 6 Patient 7 Patient 8	Patient 4 Patient 5 Patient 7	Patient 1 Patient 2 Patient 3 Patient 6 Patient 8
Days from CP transfusion to Ct value ≥ 40	1.00^a^	5.00 (2.00–7.00)	1.00^a^	5.00^b^	2.00^b^	3.00 (1.00–7.00)
virus-specific IgG titer ratio	1.20 (1.15–1.85)	1.06 (0.99–1.54)	1.15 (1.10–1.21)	1.69 (0.97–1.85)	1.44^b^	1.15 (1.00–1.45)
Length of hospital stay, days	20.00 (18.00–28.00)	32.50 (28.50–35.75)	27.50 (18.50–35.75)	28.00 (28.00–30.00)	31.50^b^	24.00 (19.00–33.00)

Data are indicated as median (IQR). The virus-specific IgG titer ratio indicates the value tested by MCLIA at day 1 after CP transfusion divided by the value before CP transfusion

*CP* convalescent plasma, *NAT50* neutralizing antibody titer which was calculated with the highest dilution of plasma that resulted in a 50% reduction of virus infection

^a^Days from CP transfusion to Ct value≥40 for all eligible patients was 1.00 day

^b^Data represented the individual or average value due to lack of clinical source

## Discussion

This retrospective observational study explored the potential efficacy and safety of CP treatment in 8 patients who were critically or severely ill with COVID-19. One or two doses of CP with a total of 300–400 mL was well tolerated by all patients without any adverse effects. Improved clinical conditions as indicated by improvement of oxygen support and chest CT imaging were observed in most patients after CP treatment. The viral load as estimated by the Ct value also declined to undetectable level within 7 days post transfusion.

It has been suggested that CP served as a method of passive immunity therapy^18,19^ could significantly reduce the mortality of patients with SARS infection.^8,9^ One possible mechanism for the efficacy of CP therapy is the NAbs from CP which may lead to the clearance of viraemia.^20,21^ Our results showed that only plasma from donor 4, 5, and 7 had relatively high neutralizing activity (NAT50 > 1:640). This is consistent with a recent finding that the majority of CP donors had relatively modest neutralizing activity and a small proportion of donors had high neutralization activity.^22^ It is not surprising since all donors were previously moderate or mild patients, and there is evidence that mild patients frequently had a lower level of SARS-CoV-2 specific antibodies than severe patients.^23^ Assessing the effects of neutralizing activity of CP on the patients’ clinical efficacy, we found that patients treated by CP with high NAT50 (>1:640) had more obvious improvement than patients receiving low NAT50 value (≤1:640) of CP, including shorter negative conservation time of viral RNA, and higher increment of IgG level after CP transfusion. In line with other publications,^14^ our results indicated that CP with high concentration of NAbs may contribute to the clearance of the virus. Based on the fact that CP donors who usually recovered from mild infection may not generate adequate protective antibodies, and the levels of plasma neutralizing activity required to prevent SARS-CoV-2 re-infection are currently unknown, more studies are necessary to assess the minimum threshold of NAb titers necessary to prevent SARS-CoV-2 reinfection.

In addition to pseudovirus based neutralization test, this study also employed multiple SARS-CoV-2 serology tests and receptor-binding assay. The results demonstrated that CP donors had a wide range of antibody levels measured across multiple platforms. Pseudotyped virus assay, an alternative of neutralization test which is considered as the optimal assay to determine the antiviral activity of antibodies, could measure how effectively donor plasma or serum can inhibit virus infection of target cells.^24^ But it is not feasible to implement neutralization test or pseudotyped virus assay as a measurement of antiviral antibodies for general population investigation. By contrast, serology tests are more convenient and practical. Here we examined the correlations between serology test results and neutralization assay in the CP samples, which is seldomly explored in other studies. Our results indicated that S-RBD and NP specific IgG ELISA titers had a significant strong correlation with NAb level, and IgG MCLIA titer showed a modest correlation with neutralization activity. However, the inhibitory activity of receptor binding of the CP samples had a low degree of association with neutralization activity. These findings may provide some clues about that ELISA or MCLIA assays may serve as a surrogate for pseudovirus neutralization assay to predict the degree of neutralization activity present in recovered patients or vaccine recipients. Studies with larger sample size are necessary to further explore these alternative serology tests which could help to refine the CP selection, as well as inform immunogenicity of vaccines against SARS-CoV-2.

The treatment timing is considered as another important factor associated with the effectiveness of CP therapy.^8^ Viraemia reaches to the peak in the first week of infection for most viral illnesses. Patients usually develops a primary immune response by days 10–14, which is followed by virus clearance.^6,7^ The largest study involved the CP treatment of 80 patients in Hong Kong with SARS found that the better clinical outcome was observed among patients who were given CP before day 14 of illness and among cases who were PCR positive and seronegative for coronavirus at the time of plasma infusion.^7^ A recent study on COVID-19 demonstrated that CP therapy could not reduce the mortality rate in critically ill patients with end-stage disease.^25^ Thus, to obtain the greatest benefit from CP, treatment should be administered early in the course of the disease (e.g., before SARS-CoV-2 seroconversion). In our study, only three patients were given CP before 21 days from illness onset, and all patients had developed SARS-CoV-2-specific IgG before CP transfusion. These three patients tended to show a more rapid negative conversion of viral RNA, and shorter hospital stays compared to other patients who were transfused after 21 days. The late administration of CP may result in the fact that the patients with critical illness and complications did not show obvious clinical improvement. Specifically, patient 1 who was given CP transfusion on the 30th day of infection, and had suffered from bacterial pneumonia prior to CP therapy, showed latest conversion of virus nucleic acid on posttransfusion day 7. On the other hand, patient 2 was observed rapid decrease of Ct value on posttransfusion day 1 and obviously promoted clinical manifestation after receiving CP early after disease onset (day 12). Our results support that CP treatment in potentially critically ill patients with COVD-19 early in the course of disease may be more effective. Most patients with severe COVID-19 were featured by substantially elevated levels of proinflammatory cytokines, which was characterized as cytokine release syndrome.^26–28^ Our study also observed abnormally high levels of proinflammatory cytokines (especially IL-6) in some patients prior to CP therapy. Notably, inflammatory cytokines, including IL-6 and TNF-α, proinflammatory/anti-inflammatory ratios (IL-6/IL-10, and IL-6/IL-4) unexpectedly kept increased within 5 days of CP treatment in almost half of patients, which was not compatible with another study on CP treatment for COVID-19.^13^ It is probably because that increased systemic cytokine production may lead to the pathophysiology of severe COVID-19, including ARDS and multiple organ failure,^28^ and it might be unable to attenuate the inflammatory damage soon after CP transfusion. Elevated IL-6 level was found to be a stable indicator of poor outcome in patients with severe COVID-19 with pneumonia and ARDS.^23^ Moreover, lymphopenia has been proven to be an effective and reliable indicator of the clinical severity in COVID-19 patients^29^ Early administration of CP containing NAbs may not only inhibit viral entry and replication, but also consequently blunt an early proinflammatory pathogenic endogenous response and restore the immune system.^4,30^ Thus, we suggest that CP be given at an early stage in patients at high risk of subsequent deterioration (for instance, persistently abnormal inflammatory cytokines, and lymphopenia) for maximizing efficacy to prevent cytokine storms. Besides, to prevent from worsening disease outcome, it is beneficial to monitor the above-mentioned prognostic biomarkers for patients at high risk of developing ARDS or multiple organ failure, especially for those with chronic diseases, such as hypertension, diabetes, and COPD.

Based on our findings, the dose of infused CP might play a role on its therapeutic effect, as demonstrated by the result that the viral nucleic acid in patients transfused with 400 mL of CP tended to turn faster to undetectable than that in patients who received 200 mL of CP. While a study about the CP therapy in SARS patients found that there was no correlation between clinical outcome and the volume of infused plasma.^7^ Future large-scale studies are needed to investigate the association between the dose of CP transfusion and its clinical efficacy.

There are some limitations that should be noted in this study. First, this study was a case series with small sample size, and the outcome of the CP treated patients was not compared with a control group of patients who did not receive the intervention. Second, the patients received other therapies (including antiviral agents, antibiotics or antifungal drugs, and corticosteroids), making it impossible to discriminate the specific contribution of CP to the clinical course or outcomes. Moreover, CP was administered 9–34 days after admission in this study. The association between the transfusion timing and clinical outcomes should be further clarified. In addition, patients in the current study were given different doses of CP. It is unclear whether the doses and the titers of antibodies were associated with the treatment efficacy. Despite these limitations, this study provided more evidence to support that CP therapy might be a promising option to treat COVID-19 patients, which is also supported by the recent issue by FDA of emergency use authorization for CP as potential promising COVID-19 treatment.^31^ Overall, this study not only provided more evidence on the potential efficacy and safety of CP therapy, but also contributed to the quality control of donated plasma and reasonable clinical application of CP transfusion.

In conclusion, our preliminary study indicated that CP might be a potential therapy for severe patients with COVID-19. We observed improvement of clinical features without the occurrence of serious adverse reactions following CP transfusion. Further well-designed randomized clinical trials are needed to evaluate the efficacy and safety of CP transfusion, and to explore best donation candidates with high virus-specific antibodies, and indications for CP therapy (e.g., optimal transfusion time point, early warning indicators, and transfused dose).

## Materials and methods

### Patients

This study was performed from February 17, 2020, to April 10, 2020, at four centers, Chongqing Public Health Medical Center, Chongqing Three Gorges Central Hospital, Yongchuan Hospital of Chongqing Medical University, and Affiliated Hospital of North Sichuan Medical College. All patients were diagnosed as critical or severe COVID-19 pneumonia according to the WHO Interim Guidance^32^ and the Guideline of Diagnosis and Treatment of COVID-19 of National Health Commission of China (version 6.0)^33^ with laboratory confirmation by real-time RT-PCR assay. Patients were enrolled to receive CP treatment if they met any of the following criteria: (1) respiratory distress (RR ≥ 30 breaths/min); (2) oxygen saturation at rest ≤93%; (3) PaO_2_/FiO_2_ ≤ 300 mmHg; (4) severe complications (e.g., respiratory failure, mechanical ventilation support, septic shock, or failure of other organs). Patients with any of the following conditions were excluded: (1) allergic history to plasma, plasma protein or sodium citrate; (2) other serious syndromes not suitable for CP transfusion, such as irreversible severe organ dysfunction. A total of 8 patients were treated with CP transfusion in the study.

This study was approved by the ethical committee of Chongqing Public Health Medical Center (approval number, 2020-030-01-KY). All patients signed a written informed consent before any procedure was carried out. If patients cannot make rational decisions, the consents were signed by their family members on behalf of the patients. This study was conducted in accordance with the Helsinki Declaration as revised 1981.

### Donors for convalescent plasma transfusion

CP was obtained from donors who had recovered from COVID-19 infection. The recovery status was defined as follows: (1) aged between 18 and 55 years; (2) at least 3 weeks following symptom onset; (3) afebrile status for at least 3 days; (3) significant improvement in respiratory symptoms; (4) two consecutively negative results of sputum SARS-CoV-2 of real-time RT-PCR assay (one-day sampling interval). Persons who met all criteria were eligible for plasma donation. Written informed consent was obtained from each donor.

### Plasma preparation

Apheresis was performed using Haemonetics MCS + LN90 00-220E blood cell separator (Haemonetics, Boston, MA, USA). Convalescence plasma for treatment was collected from 7 donors. A 200 or 400 mL of ABO-compatible plasma sample was collected from each donor, and each sample was divided and stored as 100 or 200 mL aliquots at 4 °C without any detergent or heat treatment. The CP was then treated with methylene blue and light treatment for 30 min in the medical plasma virus inactivation cabinet (Shanghai blood technology Co., Ltd, Shanghai, China).

The plasma samples were tested negative for hepatitis B virus, hepatitis C virus, HIV, syphilis, and blood type irregular antibody. As a routine check with plasma donation, the CP was also confirmed negative for residual SARS-CoV-2 by RT-PCR.

### RT-PCR detection of SARS-CoV-2 RNA

Throat swab samples were collected from patients for extracting SARS-CoV-2 RNA using the RNA Viral Kit (Daan, Guangdong, China). The real-time RT-PCR assay was performed using commercials kit specific for SARS-CoV-2 nucleic acid detection (Liferiver, Shanghai, China; Shengxiang, Sansure Biotech, Hunan, China) approved by the China National Medical Products Administration (approve numbers, 20203400057 for Liferiver, and 20203400064 for Shengxiang). Two target genes, including open reading frame1ab (ORF1ab) and nucleocapsid protein (N), were simultaneously amplified using the real-time RT-PCR assay. Each transcript provided a Ct value, which is the number of cycles required for the fluorescent signal. A higher Ct value is correlated with a lower viral load. A Ct value less than 40 was defined as a positive result, and a Ct value of 40 or more was defined as a negative test. All procedures involving clinical specimens and SARS-CoV-2 were performed in a biosafety level 3 laboratory.

### Detection of specific IgG and IgM levels against SARS-CoV-2

The collected CP and serum samples from the donors and patients were inactivated at 56 °C for 30 min and stored at −20 °C before testing, and serially diluted before determination. IgG and IgM against SARS-CoV-2 were tested using MCLIA kits supplied by Bioscience Co. (Tianjing, China) (approved by the China National Medical Products Administration; approval numbers, 20203400183 (IgG) and 20203400182 (IgM)), according to the manufacturer’s instructions. MCLIA for IgG or IgM detection was developed based on a double-antibody sandwich immunoassay. The recombinant antigens containing the nucleoprotein and a peptide from the spike protein of SARS-CoV-2 were conjugated with FITC and immobilized on anti-FITC antibody-conjugated magnetic particles. The tests were conducted on an automated magnetic chemiluminescence analyzer (Axceed 260, Bioscience, Tianjing, China) according to the manufacturer’s instructions. The MCLIA titers of specific IgG and IgM antibodies were defined as the highest dilution giving a chemiluminescence value of more than or equal to 1. All tests were performed under strict biosafety conditions.

### Detection of specific IgG levels against SARS-CoV-2 S-RBD and NP

SARS-CoV-2 NP and S-RBD specific IgG antibodies in plasma were measured by in-house ELISA separately. Purified NP and S-RBD antigens were coated onto MaxiSorp ELISA plates (Corning Costar, Acton, MA, USA) in 0.1 M carbonate buffer (pH 9·6) at concentration of 0.2 μg/mL overnight at 4 °C, respectively. Plates were washed 4 times with phosphate-buffered saline (PBS) containing 0.1% vol/vol Tween-20 (PBST) and blocked with 1% bovine serum albumin for 2 h at 37 °C. The plates were then washed with PBST. The serum samples were diluted to 80-fold into PBS as initial concentration, and then serial 2-fold diluted until 81920-fold. The serial dilutions of serum samples were added to the plate wells and incubated, followed by wash and incubation with anti-human IgG HRP-conjugated coat secondary antibody (Abcam, Cambridge, UK). After 4 washes, plates were developed by tetramethylbenzidine substrate (TianGen Biotech Co., Beijing, China) at room temperature in the dark. The absorbance was measured at 450 nm using a microplate reader (Molecular Devices Co., Sunnyvale, CA, USA) after adding the stop solution (2 M sulphuric acid). All samples were run in duplicate. The titers of NP and S-RBD specific IgG antibodies were defined as the highest dilution giving an absorbance value of more than 2.1 times that of the negative control.

### Receptor-binding assay

Inhibitory effects of the CP samples on RBD-Fc binding to receptor angiotensin-converting enzyme 2 (ACE2) were tested using an ELISA-based assay. Recombinant soluble human ACE2 (Sino Biological) was coated at 2 µg/ml to 96-well ELISA plates (Corning Costar) in 0.1 M carbonate buffer (pH 9.6) at 4 °C overnight. Plates were washed 4 times with 0.1% vol/vol PBST and blocked with 0.1% bovine serum albumin for 2 h at 37 °C. 80 ng/ml recombinant SARS-CoV-2 Spike RBD-mFc (Sino Biological) was mixed with the presence or absence of serially diluted CP or serum samples 1:1 and incubated at 37 °C for 1 h, then add the 100 μl mixed solution to the wells. Incubated at 37 °C for 10 min, 100 μl of the HRP conjugated goat anti-mouse IgG (ZSGB-BIO) was added to the wells. After incubation at 37 °C for 1 h, 100 μl of the substrate TMB was added to the wells. Developed at room temperature in the dark for 5 min, it was terminated with the stop solution (2 M sulphuric acid). The absorbance was measured at 450 nm. All samples were run in duplicate. The 50% inhibitory titer (IT50) was defined as the dilution of serum or plasma that inhibits 50% RBD-Fc binding to receptor ACE2 using a linear interpolation algorithm.

### Pseudovirus based neutralization assay

The neutralization of plasma samples was measured by a pseudovirus-based neutralizing assay as described previously.^34^ In brief, pseudovirus was incubated with serial dilutions of the plasma samples (six dilutions in a 3-fold step-wise manner) in duplicate for 1 h at 37 °C, together with the virus control and cell control wells in hexaplicate. Then, freshly Huh-7 cells (Japanese Collection of Research Bioresources [JCRB], 0403) were added to each well. Following 24 h of incubation in a 5% CO_2_ environment at 37 °C, the luminescence was measured using a microplate luminometer (PerkinElmer, Ensight). The NAb titers (NAT50) were defined as the 50% inhibitory dilution (ID50) which was calculated with the highest dilution of plasma that resulted in a 50% reduction of relative light units compared with virus control.

### Clinical data collection and efficacy assessment

Clinical information of the patients before and after CP transfusion was retrieved from the hospital electronic medical records system, including: (1) basic clinical data: age, sex, days of admission from symptom onset, presenting symptoms, comorbidities, and other treatments; (2) CP transfusion information: time and dose of CP infusion, complications prior to CP therapy, and adverse effects; (3) clinical features, laboratory data, and chest CT imaging.

Adverse events and serious adverse events associated with CP transfusion were assessed by the clinician. The primary indicators for efficacy assessment were the changes of clinical symptoms, laboratory parameters, and radiological image after CP intervention. Clinical outcomes include discharge and hospitalization.

### Statistical analysis

Continuous variables were summarized as median and IQR or range. Spearman correlation analyses were used to calculate the correlations among log_2_-transformed anti-SARS-CoV-2 specific IgG and IgM MCLIA titers, anti-S-RBD and anti-NP specific IgG ELISA titers, IT50, and NAT50 of CP. Graphs were plotted using GraphPad Prism 7.0 (GraphPad Software, San Diego, CA, USA). Correlation analysis was performed using SPSS 22.0 (SPSS Inc., Chicago, IL, USA). Two-tailed *P* value of less than 0.05 was considered statistically significant.

## Supplementary information

Supplementary Materials

## Data Availability

The authors declare that the data supporting the findings of this study are available within the paper and its Supplementary materials.
