# Supplementary material for: The efficacy assessment of convalescent plasma therapy for COVID-19 patients: a multi-center case series
Source: Signal Transduct Target Ther. 2020 Oct 6;5:219. doi: 10.1038/s41392-020-00329-x (PMC7538052; doi:10.1038/s41392-020-00329-x)
Supplement: Supplementary file 1 — Supplementary Materials [file 41392_2020_329_MOESM1_ESM.docx]

Supplementary Materials for

The efficacy assessment of convalescent plasma therapy for COVID-19 patients: a multi-center case series

Hao Zeng, Dongfang Wang, Jingmin Nie, Haoyu Liang, Jiang Gu, Anne Zhao, Lixin Xu, Chunhui Lang, Xiaoping Cui, Xiaolan Guo, Changlong Zhou, Haibo Li, Bin Guo, Jinyong Zhang, Qiang Wang, Li Fang, Wen Liu, Yishan Huang, Wei Mao, Yaokai Chen, Quanming Zou

Correspondence to: maoweicqbc@163.com; yaokaichen@hotmail.com; qmzou2007@163.com

**This PDF file includes:**

Figure S1

Table S1

Figure. S1.

Changes of some other laboratory markers before and at day 1-5 after convalescent plasma transfusion. (a) tumor necrosis factor-α (TNF-α) (normal range: 0-2.31 pg/mL). (b) interferon-γ (IFN-γ) (normal range: 0-7.42 pg/mL). (c) interleukin-2 (IL-2) (normal range: 0-5.71 pg/mL). (d) IL-10 (normal range: 0-4.91 pg/mL). (e) IL-17A (normal range: 0-20.60 pg/mL). (f) D-dimer (normal range: 0-0.55 mg/L).


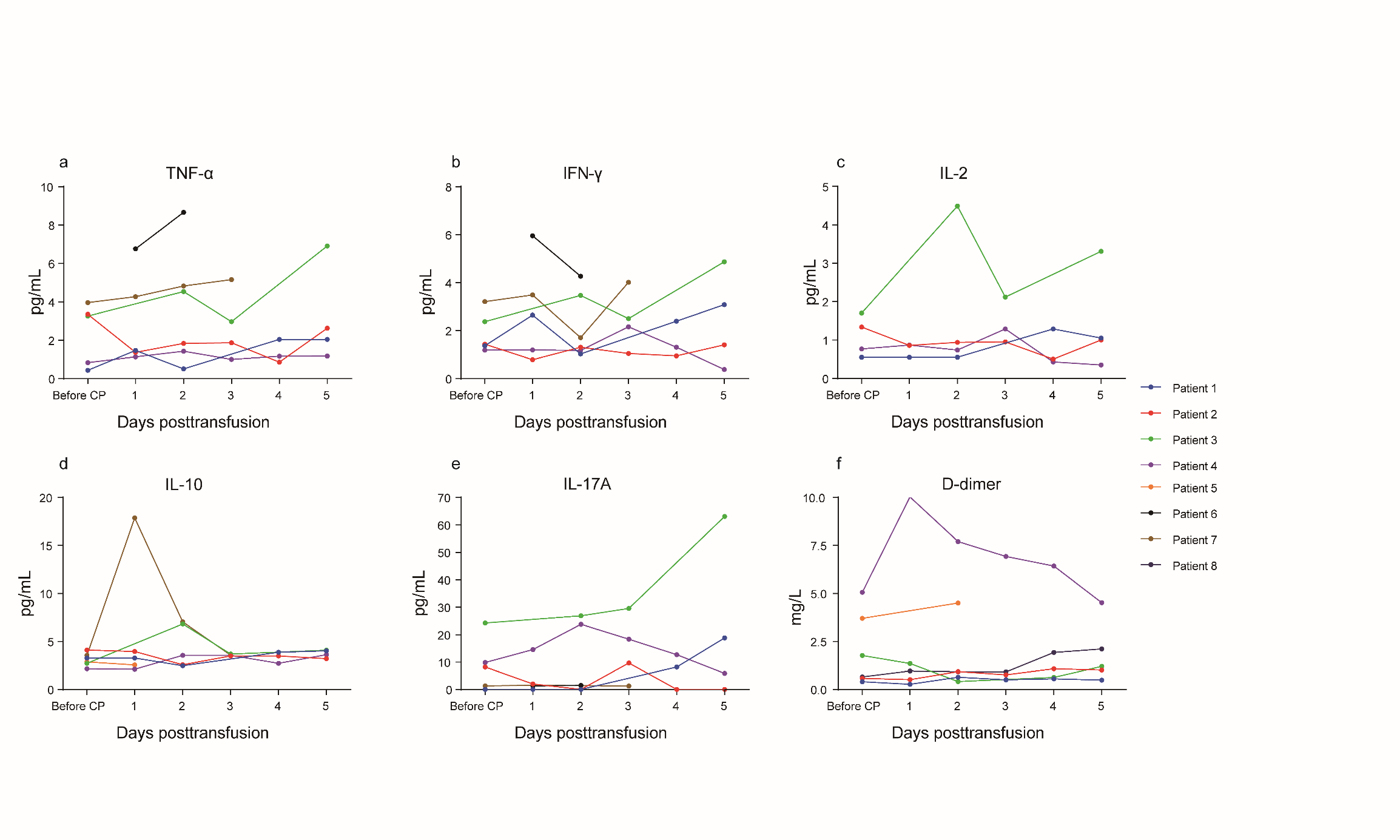


**Fig. S1**

Table S1.

Data of clinical features and laboratory markers before and after convalescent plasma transfusion.

|  | **Patients** | | | | | | | | **Median** | **1st Quartile** | **3rd Quartile** |
| --- | --- | --- | --- | --- | --- | --- | --- | --- | --- | --- | --- |
|  | **1** | **2** | **3** | **4** | **5** | **6** | **7** | **8** |  |  |  |
| **Clinical characteristics** |  |  |  |  |  |  |  |  |  |  |  |
| Body temperature, ℃ |  |  |  |  |  |  |  |  |  |  |  |
| Before CP transfusion | 36.3 | 36.2 | 36.8 | 36.4 | 36.3 | 36.6 | 36.5 | 36.5 | 36.5 | 36.3 | 36.6 |
| Day 1 | 36.3 | 36.2 | 36.2 | 37.1 | 36.9 | 36.1 | 36.9 | 36.8 | 36.6 | 36.2 | 36.9 |
| Day 2 | 36.4 | 36.5 | 36.2 | 38.5 | 36.2 | 36.6 | 37 | 36.6 | 36.6 | 36.3 | 36.9 |
| Day 3 | 36.6 | 36.2 | 36.2 | 37 | 36.5 | 36.6 | 36.6 | 36.8 | 36.6 | 36.3 | 36.8 |
| Day 4 | 36.4 | 36.2 | 36.2 | 38 | 36.5 | 36.4 | 36.7 | 36.8 | 36.5 | 36.3 | 36.8 |
| Day 5 | 36.3 | - | 36.2 | 37.7 | - | - | 36.4 | 36.5 | 36.4 | 36.3 | 37.1 |
| Respiratory rate, per min |  |  |  |  |  |  |  |  |  |  |  |
| Before CP transfusion | 19 | 19 | 21 | 27 | 20 | 19 | 14 | 28 | 19.5 | 19.0 | 25.5 |
| Day 1 | 22 | 24 | 21 | 28 | 21 | 20 | 17 | 20 | 21.0 | 20.0 | 23.5 |
| Day 2 | 20 | 19 | 20 | 20 | 21 | 22 | 21 | 21 | 20.5 | 20.0 | 21.0 |
| Day 3 | 19 | 21 | 22 | 27 | 22 | 19 | 16 | 22 | 21.5 | 19.0 | 22.0 |
| Day 4 | 19 | 20 | 21 | 20 | 22 | 23 | 18 | 20 | 20.0 | 19.3 | 21.8 |
| Day 5 | 19 | - | 20 | 30 | - | - | 19 | 20 | 20.0 | 19.0 | 25.0 |
| Heart rate |  |  |  |  |  |  |  |  |  |  |  |
| Before CP transfusion | 62 | 70 | 61 | 77 | 96 | 82 | 78 | 108 | 77.5 | 64.0 | 92.5 |
| Day 1 | 77 | 69 | 87 | 75 | 100 | 85 | 71 | 88 | 81.0 | 72.0 | 87.8 |
| Day 2 | 73 | 76 | 57 | 80 | 101 | 59 | 85 | 89 | 78.0 | 62.5 | 88.0 |
| Day 3 | 68 | 87 | 101 | 82 | 87 | 72 | 93 | 77 | 84.5 | 73.3 | 91.5 |
| Day 4 | 82 | 79 | 86 | 90 | 78 | 89 | 100 | 88 | 87.0 | 79.8 | 89.8 |
| Day 5 | 75 | - | 113 | 130 | - | - | 88 | 100 | 100.0 | 81.5 | 121.5 |
| Systolic pressure |  |  |  |  |  |  |  |  |  |  |  |
| Before CP transfusion | 119 | 117 | 100 | 130 | 145 | 101 | 126 | 117 | 118.0 | 105.0 | 129.0 |
| Day 1 | 109 | 116 | 106 | 120 | 118 | 109 | 105 | 136 | 112.5 | 106.8 | 119.5 |
| Day 2 | 116 | 114 | 102 | 130 | 116 | 122 | 116 | 141 | 116.0 | 114.5 | 128.0 |
| Day 3 | 107 | 118 | 115 | 107 | 125 | 115 | 121 | 124 | 116.5 | 109.0 | 123.3 |
| Day 4 | 106 | 108 | 107 | - | 102 | 116 | 124 | 112 | 108.0 | 106.0 | 116.0 |
| Day 5 | 111 | - | 109 | - | - | - | 126 | 125 | 118.0 | 109.5 | 125.8 |
| PaO2/FiO2 (normal range, 400-500 mL) |  |  |  |  |  |  |  |  |  |  |  |
| Before CP transfusion | 157 | 259 | 283 | 273 | - | 203 | 285 | 163 | 259.0 | 163.0 | 283.0 |
| Day 1 | 409 | 258 | 312 | 352 | - | 215 | 417 | 246 | 312.0 | 246.0 | 409.0 |
| Day 2 | 359 | 279 | - | 280 | - | - | 473 | 318 | 318.0 | 279.5 | 416.0 |
| Day 3 | 563 | 225 | 326 | 200 | - | - | - | 189 | 225.0 | 194.5 | 444.5 |
| Day 4 | 545 | - | 339 | 181 | - | - | - | 241 | 290.0 | 196.0 | 493.5 |
| Day 5 | 600 | - | 424 | 228 | - | - | - | 211 | 326.0 | 215.3 | 556.0 |
| **Laboratory results** |  |  |  |  |  |  |  |  |  |  |  |
| WBC count×109/L (normal range, 3.5-9.5) |  |  |  |  |  |  |  |  |  |  |  |
| Before CP transfusion | 8.6 | 7.2 | 9.1 | 6.8 | 5.1 | 4.3 | 7.8 | 8.0 | 7.5 | 5.5 | 8.4 |
| Day 1 | 7.1 | 6.9 | 8.8 | 7.7 | 4.7 | 8.8 | 9.3 | 13.7 | 8.3 | 7.0 | 9.2 |
| Day 2 | 7.4 | 7.8 | 5.2 | 7.4 | 5.0 | 6.5 | 8.0 | 9.5 | 7.4 | 5.5 | 7.9 |
| Day 3 | 6.4 | 6.7 | 5.0 | 10.4 | 5.2 | 6.2 | 8.2 | 17.2 | 6.6 | 5.5 | 9.9 |
| Day 4 | 6.4 | 6.9 | 5.6 | 11.7 | 4.5 | 6.5 | 8.3 | 21.8 | 6.7 | 5.8 | 10.9 |
| Day 5 | 6.1 | 6.2 | 3.9 | 9.7 | - | - | 10.1 | 22.6 | 7.9 | 5.6 | 13.2 |
| NE count×109/L (normal range, 1.8-6.3) |  |  |  |  |  |  |  |  |  |  |  |
| Before CP transfusion | 5.8 | 4.6 | 7.4 | 6.4 | 4.4 | 3.8 | 7.3 | 7.6 | 6.1 | 4.4 | 7.3 |
| Day 1 | 4.4 | 4.8 | 7.5 | 6.2 | 3.8 | 7.9 | 8.6 | 13.4 | 6.9 | 4.5 | 8.4 |
| Day 2 | 5.5 | 5.8 | 3.3 | 5.7 | 4.5 | 5.7 | 7.3 | 9.3 | 5.7 | 4.7 | 6.9 |
| Day 3 | 4.4 | 4.6 | 3.1 | 9.1 | 4.1 | 5.4 | 6.3 | 17.0 | 5.0 | 4.2 | 8.4 |
| Day 4 | 4.1 | 4.7 | 3.0 | 9.8 | 3.3 | 4.3 | 6.6 | 20.8 | 4.5 | 3.5 | 9.0 |
| Day 5 | 3.9 | 4.2 | 2.1 | 7.9 | - | - | 8.5 | 21.6 | 6.0 | 3.5 | 11.8 |
| LY count×109/L (normal range, 1.1-3.2) |  |  |  |  |  |  |  |  |  |  |  |
| Before CP transfusion | 1.8 | 1.6 | 1.3 | 0.3 | 0.2 | 0.5 | 0.3 | 0.1 | 0.4 | 0.2 | 1.5 |
| Day 1 | 1.7 | 1.3 | 0.7 | 0.9 | 0.3 | 0.8 | 0.1 | 0.3 | 0.7 | 0.3 | 1.2 |
| Day 2 | 1.1 | 1.4 | 1.2 | 0.8 | 0.3 | 0.7 | 0.2 | 0.1 | 0.8 | 0.2 | 1.2 |
| Day 3 | 1.1 | 1.5 | 1.4 | 0.6 | 0.3 | 0.7 | 0.8 | 0.1 | 0.8 | 0.4 | 1.3 |
| Day 4 | 1.3 | 1.4 | 2.0 | 1.0 | 0.3 | 1.6 | 0.8 | 0.4 | 1.1 | 0.5 | 1.5 |
| Day 5 | 1.3 | 1.3 | 1.4 | 0.9 | - | - | 0.8 | 0.4 | 1.1 | 0.7 | 1.4 |
| CRP, mg/L (normal range, <8) |  |  |  |  |  |  |  |  |  |  |  |
| Before CP transfusion | 11.4 | 7.9 | 10.5 | 234.1 | 47.3 | 7.2 | - | - | 10.9 | 7.7 | 94.0 |
| Day 1 | 20.3 | 11.7 | 7.7 | 82.5 | 8.4 | < 10.00 | - | - | 11.7 | 8.1 | 51.4 |
| Day 2 | 41.7 | 8.7 | 10.3 | 62.9 | 70.7 | - | - | - | 41.7 | 9.5 | 66.8 |
| Day 3 | 43.3 | 6.4 | 6.3 | 128.8 | - | - | - | - | 24.9 | 6.3 | 107.4 |
| Day 4 | 39.8 | 5.3 | - | 113.6 | 8.4 | - | - | - | 24.1 | 6.1 | 95.2 |
| Day 5 | 41.9 | 5.0 | - | 58.1 | - | - | - | - | 41.9 | 5.0 | 58.1 |
| PCT, ng/mL (normal range, <0.1) |  |  |  |  |  |  |  |  |  |  |  |
| Before CP transfusion | - | - | ＜0.02 | 0.43 | 0.23 | 0.05 | 0.07 | - | 0.15 | 0.06 | 0.38 |
| Day 1 | - | 0.06 | 0.22 | 0.12 | 0.17 | 0.03 | 0.05 | - | 0.09 | 0.05 | 0.18 |
| Day 2 | - | 0.05 | 0.09 | 0.05 | 0.18 | - | 0.05 | - | 0.05 | 0.05 | 0.13 |
| Day 3 | - | 0.03 | 0.03 | 0.14 | - | - | 0.05 | - | 0.04 | 0.03 | 0.12 |
| Day 4 | - | 0.02 | 0.01 | 0.11 | - | - | 0.06 | - | 0.04 | 0.01 | 0.09 |
| Day 5 | - | 0.01 | 0.01 | 0.11 | - | - | - | - | 0.01 | 0.01 | 0.11 |
| IL-2, pg/ml (normal range, 0-5.71) |  |  |  |  |  |  |  |  |  |  |  |
| Before CP transfusion | 0.6 | 1.3 | 1.7 | 0.8 | - | - | - | - | 1.1 | 0.6 | 1.6 |
| Day 1 | 0.6 | 0.9 | - | 0.9 | - | - | - | - | 0.9 | 0.6 | 0.9 |
| Day 2 | 0.6 | 0.9 | 4.5 | 0.7 | - | - | - | - | 0.8 | 0.6 | 3.6 |
| Day 3 | - | 1.0 | 2.1 | 1.3 | - | - | - | - | 1.3 | 1.0 | 2.1 |
| Day 4 | 1.3 | 0.5 | - | 0.4 | - | - | - | - | 0.5 | 0.4 | 1.3 |
| Day 5 | 1.1 | 1.0 | 3.3 | 0.4 | - | - | - | - | 1.0 | 0.5 | 2.7 |
| IL-4, pg/ml (normal range, 0-2.80) |  |  |  |  |  |  |  |  |  |  |  |
| Before CP transfusion | 0.2 | 1.2 | 2.0 | 0.9 | - | 2.0 | 2.3 | - | 1.6 | 0.7 | 2.1 |
| Day 1 | 0.2 | 0.4 | - | 0.5 | - | 2.6 | 2.2 | - | 0.5 | 0.3 | 2.4 |
| Day 2 | 0.7 | 1.4 | 3.5 | 0.7 | - | - | 3.1 | - | 1.4 | 0.7 | 3.3 |
| Day 3 | - | 0.1 | 1.9 | 1.1 | - | - | 2.3 | - | 1.5 | 0.4 | 2.2 |
| Day 4 | 1.1 | 0.4 | - | 0.2 | - | - | - | - | 0.4 | 0.2 | 1.1 |
| Day 5 | 0.5 | 0.7 | 6.6 | 0.8 | - | - | - | - | 0.7 | 0.6 | 5.1 |
| IL-6, pg/ml (normal range, 0-5.30) |  |  |  |  |  |  |  |  |  |  |  |
| Before CP transfusion | 1.1 | 5.2 | 4.0 | 10.4 | 56.2 | 19.0 | 0.0 | 29.4 | 7.8 | 1.8 | 26.8 |
| Day 1 | 1.1 | 6.6 | - | 10.5 | 20.6 | 0.0 | 12.8 | 11.6 | 10.5 | 1.1 | 12.8 |
| Day 2 | 32.8 | 5.1 | 12.7 | 73.1 | 3.7 | - | 29.4 | 6.5 | 12.7 | 5.1 | 32.8 |
| Day 3 | - | 4.4 | 4.2 | 108.8 | - | - | 11.6 | 27.3 | 11.6 | 4.3 | 68.0 |
| Day 4 | 5.5 | 3.9 | - | 63.9 | - | - | - | 41.7 | 23.6 | 4.3 | 58.3 |
| Day 5 | 5.3 | 5.8 | 6.6 | 41.8 | - | - | - | 29.4 | 6.6 | 5.5 | 35.6 |
| IL-10, pg/ml (normal range, 0-4.91) |  |  |  |  |  |  |  |  |  |  |  |
| Before CP transfusion | 3.3 | 4.1 | 2.7 | 2.2 | - | 2.9 | 3.6 | - | 3.1 | 2.6 | 3.7 |
| Day 1 | 3.3 | 4.0 | - | 2.1 | - | 2.6 | 17.9 | - | 3.3 | 2.3 | 10.9 |
| Day 2 | 2.5 | 2.6 | 6.8 | 3.6 | - | - | 7.0 | - | 3.6 | 2.5 | 6.9 |
| Day 3 | - | 3.5 | 3.7 | 3.6 | - | - | 3.6 | - | 3.6 | 3.5 | 3.7 |
| Day 4 | 3.9 | 3.5 | - | 2.7 | - | - | - | - | 3.5 | 2.7 | 3.9 |
| Day 5 | 4.0 | 3.2 | 4.1 | 3.6 | - | - | - | - | 3.8 | 3.3 | 4.1 |
| IL-17A, pg/ml (normal range, 0-20.60) |  |  |  |  |  |  |  |  |  |  |  |
| Before CP transfusion | 0.0 | 8.2 | 24.3 | 9.8 | - | - | 1.3 | - | 8.2 | 0.7 | 17.0 |
| Day 1 | 0.0 | 2.0 | - | 14.6 | - | 1.3 | 1.5 | - | 1.5 | 0.7 | 8.3 |
| Day 2 | 0.0 | 0.0 | 26.9 | 23.8 | - | 1.5 | 1.4 | - | 1.5 | 0.0 | 24.5 |
| Day 3 | - | 9.7 | 29.5 | 18.4 | - | - | 1.3 | - | 14.0 | 3.4 | 26.7 |
| Day 4 | 8.2 | 0.0 | - | 12.7 | - | - | - | - | 8.2 | 0.0 | 12.7 |
| Day 5 | 18.8 | 0.0 | 63.1 | 5.9 | - | - | - | - | 12.3 | 1.5 | 52.0 |
| TNF-α, pg/ml (normal range, 0-2.31) |  |  |  |  |  |  |  |  |  |  |  |
| Before CP transfusion | 0.4 | 3.4 | 3.3 | 0.8 | - | - | 4.0 | - | 3.3 | 0.6 | 3.7 |
| Day 1 | 1.5 | 1.4 | - | 1.1 | - | 6.8 | 4.3 | - | 1.5 | 1.3 | 5.5 |
| Day 2 | 0.5 | 1.8 | 4.5 | 1.4 | - | 8.7 | 4.8 | - | 3.2 | 1.2 | 5.8 |
| Day 3 | - | 1.9 | 3.0 | 1.0 | - | - | 5.2 | - | 2.4 | 1.2 | 4.6 |
| Day 4 | 2.1 | 0.9 | - | 1.2 | - | - | - | - | 1.2 | 0.9 | 2.1 |
| Day 5 | 2.1 | 2.6 | 6.9 | 1.2 | - | - | - | - | 2.3 | 1.4 | 5.8 |
| IFN-γ, pg/ml (normal range, 0-7.42) |  |  |  |  |  |  |  |  |  |  |  |
| Before CP transfusion | 1.4 | 1.4 | 2.4 | 1.2 | - | - | 3.2 | - | 1.4 | 1.3 | 2.8 |
| Day 1 | 2.7 | 0.8 | - | 1.2 | - | 6.0 | 3.5 | - | 2.7 | 1.0 | 4.7 |
| Day 2 | 1.0 | 1.3 | 3.5 | 1.2 | - | 4.3 | 1.7 | - | 1.5 | 1.1 | 3.7 |
| Day 3 | - | 1.1 | 2.5 | 2.2 | - | - | 4.0 | - | 2.3 | 1.3 | 3.6 |
| Day 4 | 2.4 | 1.0 | - | 1.3 | - | - | - | - | 1.3 | 1.0 | 2.4 |
| Day 5 | 3.1 | 1.4 | 4.9 | 0.4 | - | - | - | - | 2.2 | 0.6 | 4.4 |
| ALT, u/L (normol range, 5-40) |  |  |  |  |  |  |  |  |  |  |  |
| Before CP transfusion | 13.0 | 20.0 | 18.0 | 33.0 | 11.0 | 22.2 | 51.7 | 79.3 | 21.1 | 14.3 | 47.0 |
| Day 1 | 16.0 | 22.0 | 17.0 | 38.0 | - | 19.3 | 49.5 | 136.6 | 22.0 | 17.0 | 49.5 |
| Day 2 | 13.0 | 24.0 | 19.0 | 46.0 | 12.0 | 20.0 | 50.1 | 169.2 | 22.0 | 14.5 | 49.1 |
| Day 3 | 14.0 | 23.0 | 18.0 | 25.0 | - | 19.3 | 51.7 | 143.0 | 23.0 | 18.0 | 51.7 |
| Day 4 | 18.0 | 22.0 | 14.0 | 32.0 | 15.0 | 18.7 | 42.9 | 150.5 | 20.4 | 15.8 | 40.2 |
| Day 5 | 14.0 | 17.0 | 14.0 | 43.0 | - | - | 38.5 | 131.0 | 27.8 | 14.0 | 65.0 |
| AST, u/L (mormol range, 13-35) |  |  |  |  |  |  |  |  |  |  |  |
| Before CP transfusion | 19.0 | 17.0 | 16.0 | 23.0 | 14.0 | 21.4 | 33.4 | 42.5 | 20.2 | 16.3 | 30.8 |
| Day 1 | 19.0 | 19.0 | 15.0 | 22.0 | - | 16.4 | 25.0 | 102.1 | 19.0 | 16.4 | 25.0 |
| Day 2 | 15.0 | 18.0 | 18.0 | 29.0 | 13.0 | 16.9 | 30.7 | 77.0 | 18.0 | 15.5 | 30.3 |
| Day 3 | 16.0 | 16.0 | 18.0 | 20.0 | - | 14.3 | 32.3 | 45.8 | 18.0 | 16.0 | 32.3 |
| Day 4 | 17.0 | 18.0 | 15.0 | 17.0 | 16.0 | 13.3 | 26.0 | 47.7 | 17.0 | 15.3 | 24.0 |
| Day 5 | 14.0 | 14.0 | 14.0 | 28.0 | - | - | 22.8 | 50.2 | 18.4 | 14.0 | 33.6 |
| TBIL, μmol/L (normal range, 0 -26) |  |  |  |  |  |  |  |  |  |  |  |
| Before CP transfusion | 27.4 | 17.9 | 17.1 | 19.7 | 4.6 | 3.9 | 14.7 | 26.4 | 17.5 | 7.1 | 24.7 |
| Day 1 | 14.1 | 9.5 | 10.4 | 15.4 | - | 6.6 | 6.3 | 16.6 | 10.4 | 6.6 | 15.4 |
| Day 2 | 15.9 | 9.9 | 10.9 | 20.7 | 5.3 | 4.9 | 7.4 | 15.6 | 10.4 | 5.8 | 15.8 |
| Day 3 | 10.5 | 9.2 | 12.0 | 29.5 | - | 6.8 | 12.7 | 14.2 | 12.0 | 9.2 | 14.2 |
| Day 4 | 11.0 | 8.2 | 9.7 | 23.8 | 2.8 | 5.7 | 11.3 | 18.4 | 10.4 | 6.3 | 16.6 |
| Day 5 | 11.7 | 9.3 | 9.7 | 30.2 | - | - | 9.9 | 22.7 | 10.8 | 9.6 | 24.6 |
| PT, second (normal range, 11-14) |  |  |  |  |  |  |  |  |  |  |  |
| Before CP transfusion | 11.1 | 11.4 | 10.8 | 14.9 | 11.5 | - | - | 10.1 | 11.3 | 10.6 | 12.4 |
| Day 1 | 11.3 | 11.5 | 10.8 | 15.1 | - | - | - | 9.4 | 11.3 | 10.1 | 13.3 |
| Day 2 | 10.4 | 10.8 | 9.0 | 15.7 | 12.0 | - | - | 9.6 | 10.6 | 9.5 | 12.9 |
| Day 3 | 10.4 | 10.7 | 9.2 | 17.0 | - | - | - | 9.9 | 10.4 | 9.6 | 13.9 |
| Day 4 | 10.5 | 10.5 | 9.7 | 16.2 | - | - | - | 9.5 | 10.5 | 9.6 | 13.4 |
| Day 5 | 10.2 | 10.2 | 11.8 | 16.3 | - | - | 11.6 | 9.6 | 10.9 | 10.1 | 12.9 |
| D-dimer, mg/L (normal range, 0-0.05) |  |  |  |  |  |  |  |  |  |  |  |
| Before CP transfusion | 0.4 | 0.6 | 1.8 | 5.1 | 3.7 | - | - | 0.7 | 1.2 | 0.5 | 4.0 |
| Day 1 | 0.3 | 0.5 | 1.4 | 10.0 | - | - | - | 1.0 | 1.0 | 0.4 | 5.7 |
| Day 2 | 0.6 | 0.9 | 0.4 | 7.7 | 4.5 | - | - | 0.9 | 0.9 | 0.6 | 5.3 |
| Day 3 | 0.5 | 0.8 | - | 6.9 | - | - | - | 0.9 | 0.8 | 0.6 | 5.4 |
| Day 4 | 0.6 | 1.1 | 0.6 | 6.4 | - | - | - | 1.9 | 1.1 | 0.6 | 4.2 |
| Day 5 | 0.5 | 1.0 | 1.2 | 4.5 | - | - | - | 2.1 | 1.2 | 0.8 | 3.3 |
